# The IVIG treatment response in autoimmune polyendocrine syndromes type 2 with anti-GAD65 antibody-associated stiff person syndrome: a case report and literature review

**DOI:** 10.3389/fimmu.2024.1471115

**Published:** 2025-01-07

**Authors:** Yulong Yang, Hailin Jiang, Wenming Yang, Han Wang, Meixia Wang, Xiang Li, Peng Huang, Shuzhen Fang, Wenjie Hao, Yue Yang, Furong Zhao, Wei He

**Affiliations:** ^1^ Department of Neurology, The First Affiliated Hospital of Anhui University of Traditional, Chinese Medicine, Hefei, Anhui, China; ^2^ Key Laboratory of Xin’An Medicine, Ministry of Education Hefei, Anhui, China; ^3^ Center for Xin’an Medicine and Modernization of Traditional Chinese Medicine, Institute of Health and Medicine Hefei Comprehensive National Science Center, Hefei, Anhui, China

**Keywords:** anti-GAP65 antibody, stiff person syndrome, autoimmune polyendocrine syndrome type 2, intravenous immunoglobulin, case report

## Abstract

Autoimmune polyendocrine syndromes (APS) is a rare group of disorders caused by impaired function of multiple endocrine glands due to disruption of immune tolerance. Of which, type 2 (APS-2) is the most common. Glutamic acid decarboxylase (GAD) is the rate-limiting enzyme for the synthesis of gamma-aminobutyric acid (GABA). Anti-GAD antibodies are associated with various neurological disorders, including stiff person syndrome (SPS). SPS is characterized by axial muscle stiffness, rigidity, and intermittent painful muscle spasms, with a prevalence of one to two in a million, making it an extremely rare neurological disorder. The comorbidity of APS-2 with SPS is even rarer. Most practicing neurologists encounter only one or two cases of APS-2 combined with anti-GAD65 antibody-associated SPS in their careers, resulting in underdiagnosis and undertreatment, leading to severe disability and suffering. This case report describes a young male who initially exhibited hair loss, vitiligo, and previously unreported eosinophilia. Before his diagnosis, he was admitted multiple times, with symptoms improving following the addition of intravenous immunoglobulin (IVIG) therapy to a poor treatment regimen. This paper aims to increase physicians’ awareness of this condition, enhancing the likelihood of early diagnosis and treatment.

## Introduction

Autoimmune polyendocrine syndromes (APS) is rare clinical conditions caused by immune tolerance disruption, leading to impaired function of multiple endocrine glands and ultimately causing functional damage. APS can be classified into three main types: APS-1, APS-2, and X-linked immunodysregulation, polyendocrinopathy, and enteropathy (IPEX) (1). APS-1 is an autosomal recessive disorder caused by mutations in the autoimmune regulator gene (AIRE). APS-2 is a complex genetic disease involving multiple genes, with genetic susceptibility associated with human leukocyte antigen (HLA), major histocompatibility complex-related gene A, lymphotyrosine phosphatase, and cytotoxic T cell-associated antigen 4 gene polymorphisms. IPEX is an X-linked recessive disorder caused by mutations in the *JM2* gene, located in the centromere region of the X chromosome (Xq11.3-q13.3), later known as forkhead box protein 3 (FOXP3). IPEX affects only males, often presenting with refractory diarrhea, diabetes, eczema, hemolytic anemia, thyroiditis, and severe infections within a few months of birth (2, 3).

Glutamic acid decarboxylase (GAD) is an intracellular enzyme expressed in neurons and insulin-secreting pancreatic β cells. Its physiological function is the decarboxylation of glutamate to gamma-aminobutyric acid (GABA) (4, 5). Previous studies have shown that GAD65 is associated with various diseases, including stiff person syndrome (SPS) (6–8).

SPS was first reported by Woltman and Moersch in 1956, who described 14 patients with this rare neuroimmune disease characterized by progressive axial muscle stiffness and painful muscle spasms (9). The pathophysiology of SPS is not completely understood but is believed to involve anti-glutamic acid decarboxylase (anti-GAD) antibodies. High serum anti-GAD65 antibody titers (>1:10,000 by ELISA) are found in up to 80% of patients (10, 11). Treatment typically involves GABAergic drugs such as benzodiazepines, corticosteroids, plasmapheresis, and intravenous immune globulin (12).

The incidence of SPS is extremely low. According to a survey by Hadavi et al., the incidence in the general population of the United Kingdom is about 1 in 1,000,000, with females affected approximately twice as often as males (13). The incidence rate in Asia and other regions has not been reported.

APS with anti-GAD65 antibody-associated SPS are clinically rare, and almost no reports of their coexistence have been found in PubMed. Here, we present a case of APS-2 with anti-GAD65 antibody-associated SPS and review relevant literature to enhance clinical awareness of this condition.

## Case presentation

A 22-year-old man was admitted to the hospital due to recurrent episodes of consciousness loss for 10 years, which occurred twice in the past week. Ten years ago, the patient was discovered by family members lying in bed and panting heavily while sleeping. At that time, he couldn’t breathe properly and was frothing at the mouth. After 2-3 minutes, the symptoms resolved on their own. The patient was diagnosed with epilepsy at a local hospital and treated accordingly, though specific details are unknown. One month later, he experienced another loss of consciousness, accompanied by involuntary limb convulsions, upward eyeball movements, and frothing at the mouth, which improved after 3-4 minutes. These episodes recurred multiple times, leading to a diagnosis of epilepsy at another hospital. He was prescribed lamotrigine (100mg in the morning), clonazepam (4mg in the evening), and topiramate (100mg in the morning and evening). The seizures varied, predominantly presenting as minor and absent seizures, with fewer major seizures and decreasing intervals between episodes (from once a month to once every half month), including two seizures in the past week. For further diagnosis and treatment, he sought medical attention at our department, planning to address epilepsy on March 20, 2024.

**Figure 1:**
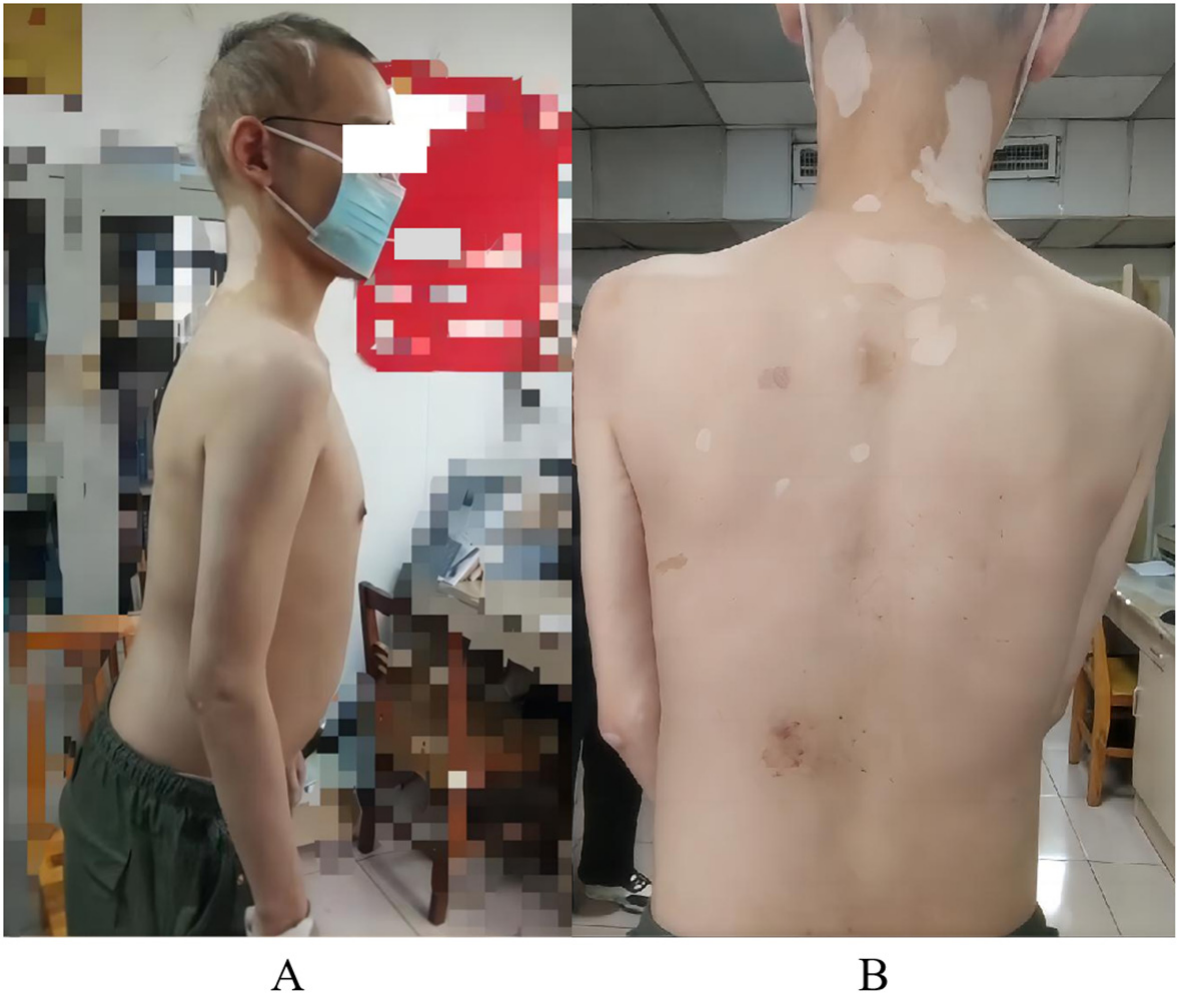
Patient’s physical examination results. **(A)** hair loss and upper body forward leaning due to torso stiffness; **(B)** multiple patchy vitiligo.

During the disease course, the patient has experienced consciousness disorders, involuntary limb shaking, frothing at the mouth, trunk stiffness leading to a hunchback (**Figure 1A**), and lower back and abdominal pain. Emotional tension often triggers involuntary abdominal muscle contractions (**Supplementary Video 1**), which disappear during sleep. His diet, sleep, and urination remain normal, though he frequently experiences diarrhea.

### Past medical history

The patient experienced hair loss in 2006, currently resulting in sparse hair (**Figure 1A**). He developed vitiligo in 2007, which remains untreated (**Figure 1B**). In 2015, eosinophilia was detected during treatment for diarrhea; he is currently maintained on 10 mg acetate prednisone daily. In 2023, his blood sugar rose to 21.38 mmol/L (3.9-6.1), leading to a diagnosis of type I diabetes. He administers 14 IU insulin glargine nightly, 11 IU insulin aspart in the morning and noon, and 14 IU in the evening, along with 0.25 g metformin nightly, though blood sugar control remains poor. Upon admission, he was found to have electrolyte disorders, particularly hypokalemia. He has been taking 1 g potassium chloride sustained-release tablets three times a day, but electrolyte levels remained abnormal. In November 2023, autoimmune thyroid disease (AITD) was diagnosed with high anti-thyroglobulin and anti-thyroid peroxidase antibodies. Based on the above history, the patient was initially diagnosed with APS by other hospitals in November 2023. His parents are both healthy with no history of neurological, autoimmune, or genetic diseases, but an uncle has a history of vitiligo.

### Physical examination

The patient had a clear mind and clear language, though slightly delayed reactions. He exhibited body stiffness, hunchback, upper body leanness, multiple patchy white spots on the skin, sparse hair, normal muscle strength and tension in the limbs, normal tendon reflexes, no meningeal irritation signs, and no pathological signs detected.

### Diagnostic workup

**Figure 2:**
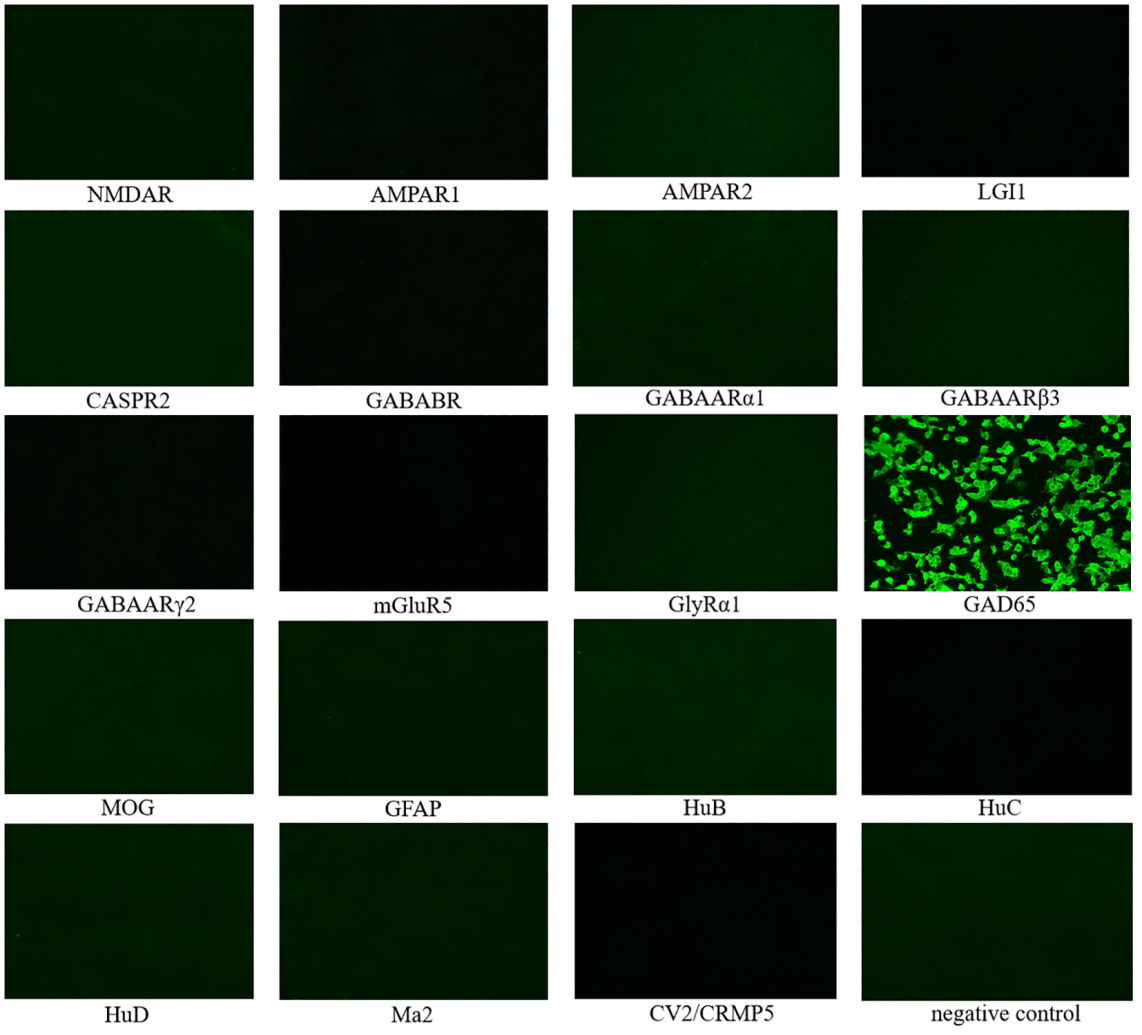
19 CSF autoimmune-related antibody tests showed positive results for anti-GAD65 antibody, while the rest were negative. NMDAR, N-methyl-D-aspartate receptor; AMPAR1, α-Amino-3-hydroxy-5-methyl-4-isoxazolepropionic acid receptor 1; AMPAR2, α-Amino-3-hydroxy-5-methyl-4-isoxazolepropionic acid receptor 2; LGI1, leucine-rich glioma inactivated 1; CASPR2, contactin-associated protein 2; GABABR,γ-Aminobutyric acid type B receptor; GABAARα1: γ-Aminobutyric acid A receptor alpha 1; GABAARβ3: γ-Aminobutyric acid A receptor beta 3; GABAARγ2: γ-Aminobutyric acid A receptor gamma 2; mGluR5, metabotropic Glutamate receptor 5; GlyRα1, Glycine receptor alpha 1; GAD65, glutamic acid decarboxylase 65; MOG, myelin oligodendrocyte glycoprotein; GFAP, glial fibrillary acidic protein; HuB, anti-neuronal nuclear antibody type-1B; HuC, anti-neuronal nuclear antibody type-1C; HuD, anti-neuronal nuclear antibody type-1D; CRMP5, collapsing response mediator proteins 5.

Cerebrospinal fluid (CSF) autoimmune-related antibody tests showed positive results for anti-GAD65 antibody (**Figure 2**). The eosinophil count was 0.93 x10^9^/L (0.02-0.52), and the percentage was 10.40% (0.4-8). Potassium was 3.47 mmol/L (3.5-5.3). Glycated hemoglobin A1c was 6.52% (4.3-6.1). Progesterone was 0.52 nmol/L (0.89-3.88). CSF routine and biochemical indicators showed glucose at 3.99 mmol/L (2.2-3.9) and CSF protein at 717.90 mg/L (80-430), with a positive Pan’s test. Other tests, including CSF neococcal smear, TORCH series, tuberculosis smear, general bacterial smear (Gram staining), general bacterial culture, hypersensitive C-reactive protein, erythrocyte sedimentation rate, anti-neutrophil cytoplasmic antibody spectrum, autoantibodies, vitamin B12, blood concentrations of lamotrigine, clonazepam, and topiramate, parathyroid hormone, free prostate-specific antigen test FPSA, tumor markers, liver and kidney function, and stool tests, showed no significant abnormalities. Furthermore, no significant abnormalities were found in the electrocardiogram, chest CT, CT scan of the entire abdomen, and cranial MRI examinations. Whole exome gene testing revealed that no point mutations (SNVs) or large segment deletions (CNVs) highly correlated with APS or with sufficient evidence of pathogenicity.

### Electromyography

**Figure 3:**
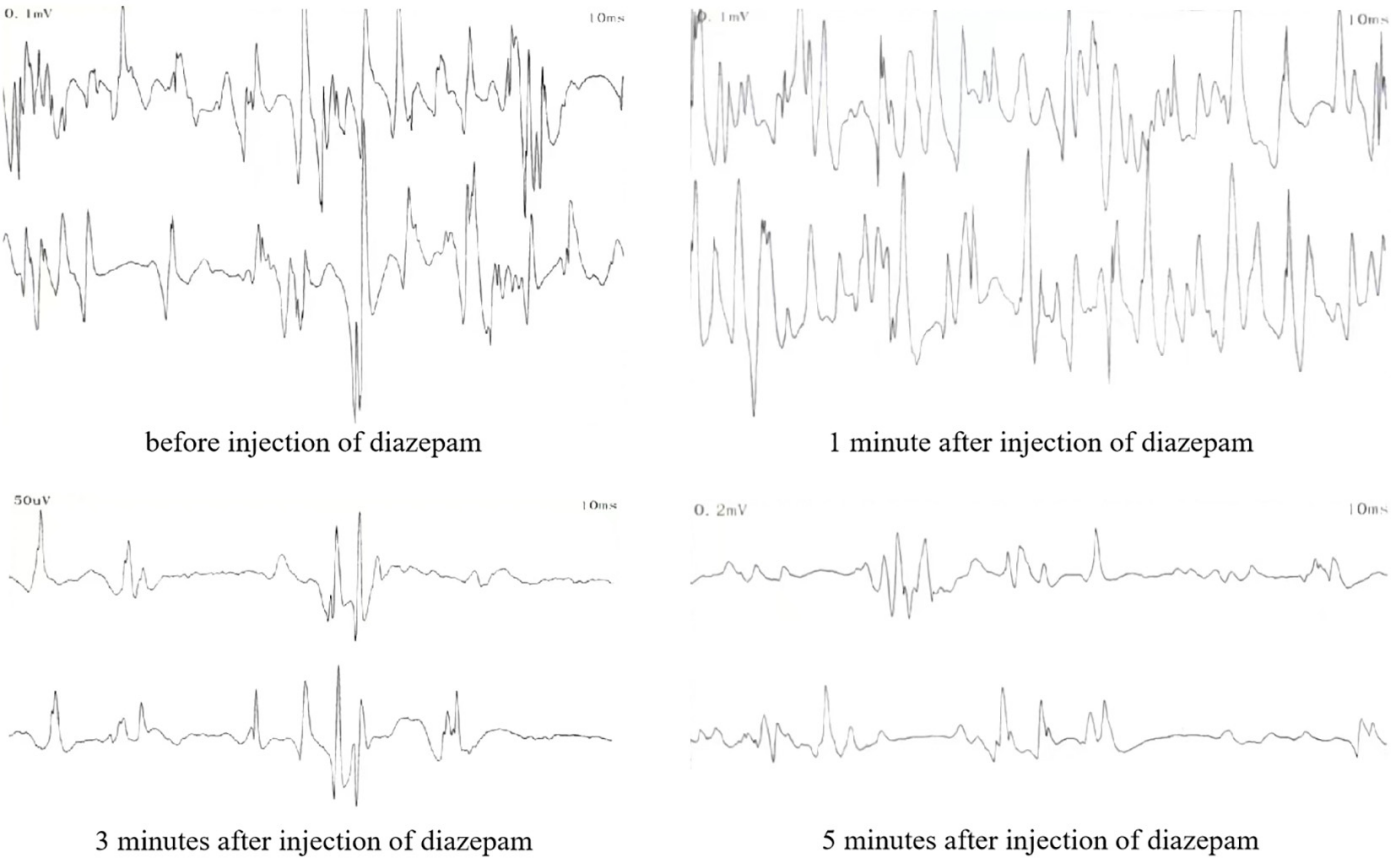
Resting potential electromyographic findings of the paraspinal muscles after intravenous injection of diazepam.

Before medication, the resting potential of the left medial femoral head muscle showed 2 to 3 positive sharp waves. The resting state of the trunk muscles showed sustained normal action potentials. After 1 minute of medication (intravenous injection of 10 mg diazepam), the resting state action potential did not decrease significantly. After 3 minutes, the action potential significantly decreased, and after 5 minutes, it significantly decreased but did not completely disappear (**Figure 3**).

**Figure 4:**
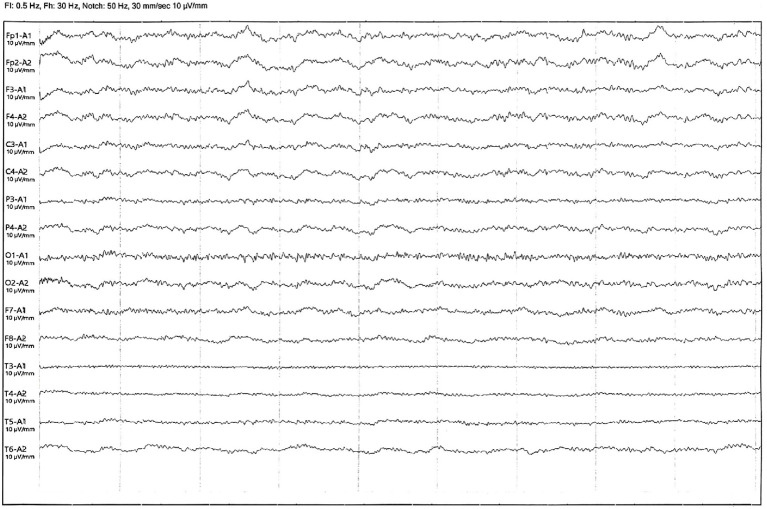
Video electroencephalography after treatment with additional IVIG.

The patient was diagnosed with autoimmune polyendocrine syndrome type II combined with anti-GAD65 antibody-associated SPS. This patient presented with hair loss as the initial symptom and was misdiagnosed for a long time. Subsequently, multiple symptoms emerged, leading to a gradual deterioration of the clinical condition. On this occasion, he was admitted to the hospital with epilepsy and had received long-term treatment with benzodiazepines, steroids, and symptomatic drugs before admission, but the efficacy was not satisfactory. Considering the treatment strategy of SPS and anti-GAD65 antibody, it was finally decided to add intravenous immunoglobulin (IVIG) (0.4 g/kg) to the existing APS treatment regimen for a period of 7 days of immunomodulatory therapy. After 3 days of treatment, the patient’s abdominal muscle contractions ceased, and the lower back and abdominal pain decreased. After 7 days of treatment, physical stiffness significantly improved, and movement clumsiness also improved. Epileptic seizures did not recur within this period, and a video electroencephalogram (VEEG) indicated mild abnormalities without epileptic discharge (**Figure 4**). The results of the treatment indicate that the disease is immune-mediated. In addition, the patient presented with epilepsy, limb stiffness, glucose metabolism disorders, electrolyte imbalances, and diarrhea. The ongoing treatment plan involves continued antiepileptic medication, measures to improve muscle stiffness, blood sugar control, electrolyte balance maintenance, and regulation of intestinal microbiota The patient was in stable condition and was discharged from the hospital.

A follow-up after one month revealed that the patient maintained overall stability, with no recurrence of seizures, involuntary abdominal muscle contractions, stiffness, or lower back and abdominal pain. Additionally, there was a significant improvement in movement clumsiness. The patient refused to be treated with IVIG again due to economic factors. Follow-up at 3 months showed that the patient was doing well, with no recurrence of epileptic symptoms and a slight improvement in clumsiness of movement compared to the situation at 1 month of discharge. At 6 months of follow-up the patient’s condition was basically good, with no significant change in her overall condition compared to her previous condition at the time of her discharge from the hospital at 3 months.

## Discussion

APS-2 is a disease that involves multiple systems and is influenced by genetic and environmental factors. Its course of disease progression varies greatly, with diverse manifestations, and its severity is influenced by multiple factors (14). Currently, there is no effective cure for APS-2, and treatment mainly relies on hormone replacement and symptomatic supportive treatment (15).

GAD65 is one of the two subtypes of GAD, encoded by the *GAD2* gene on chromosome 10 (10p12.1). It is primarily expressed at the post-natal stage and is responsible for the rapid synthesis of GABA required for synaptic transmission (16, 17). GAD65 is mainly located in the pre-synaptic end of nerve terminals, where it exists in its inactive form, unbound to the pyridoxal phosphate (PLP) cofactor. It can quickly switch from the inactive to the active form, allowing the rapid synthesis of GABA when needed (18, 19). GAD65 antibodies can cause various neurological syndromes, including borderline encephalitis, SPS, refractory epilepsy, and cerebellar ataxia (20, 21). Additionally, anti-GAD65-positive patients can exhibit myasthenia gravis, thyroiditis, pernicious anemia, vitiligo, type 1 diabetes, and other diseases (22–26).

This patient, a young male, presented with epilepsy symptoms and had comorbid conditions, including type 1 diabetes, vitiligo, and autoimmune thyroid disease, indicating the presence of autoimmune and endocrine system diseases. APS can be diagnosed when a patient exhibits dysfunction in two or more endocrine glands (27). APS-2 is the most common type, typically occurring in adults with a male-to-female ratio of 1:3 (28–30). The diagnosis of APS-2 requires the presence of two out of the following three autoimmune diseases: Addison’s disease, AITD, and type 1 diabetes (31). AITD combined with type 1 diabetes is the most common combination, accounting for about 60% of cases. This patient presented with type 1 diabetes and autoimmune thyroid disease, meeting the criteria for APS-2. Whole exome gene testing did not reveal any SNVs or CNVs highly correlated with APS and with sufficient evidence of pathogenicity. As a result, the patient was eventually recognized as having the APS-2 (AITD, type 1 diabetes) subtype of APS. Upon admission, the CSF anti-GAD65 antibody test was positive, and there were no significant abnormalities in tumor markers. The patient exhibited a stiff trunk, involuntary abdominal muscle contractions triggered by emotional tension, and symptoms that disappeared during sleep. Electromyography (EMG) showed sustained normal action potentials in trunk muscles at rest and increased motor units after passive exercise. These findings are consistent with the new diagnostic criteria for SPS proposed by Nicholas H et al. (32) in 2023, which emphasize the importance of antibodies in disease diagnosis. The diagnostic criteria for SPS are as follows: 1. Symptoms meeting one of the following criteria: a. Stiffness involving central trunk muscles and/or limb muscles; b. Episodic spasms induced by sound, sensation, or emotion involving mid-axial trunk muscles and/or limb muscles. 2. Signs meeting one of the following criteria: a. Increased muscle tone (trunk or extremities); b. Synchronized stiffness of paraspinal and rectus abdominis muscles; c. Lumbar lordosis deformity. 3. Serology meeting one of the following criteria: a. High titer anti-GAD65 antibody in serum or positive anti-GAD antibody in CSF; b. Positive serum and/or CSF anti-GlyR antibody; c. Positive serum and/or CSF anti-amphiphysin antibodies; 4. Electrophysiology meeting one of the following criteria: a. Needle pole electromyography indicating improper relaxation of paraspinal muscles; b. Surface EMG recording overreaction to sound or external sensations; c. Co-contraction of agonist-antagonist muscles detected by EMG. 5. Exclusion of other diagnoses. The patient met all the diagnostic criteria for SPS. Thus, the comprehensive diagnosis was anti-GAD65 antibody-associated SPS and APS-2 (AITD, type 1 diabetes). However, after intravenous injection of 10 mg diazepam, the effect was slow, and the sustained action potential in the resting state did not completely disappear after 5 minutes. This might be due to decreased sensitivity to diazepam from the long-term use of the antiepileptic drug clonazepam.

Although APS is called an autoimmune polyendocrine syndrome, non-endocrine organs are often affected, such as hair loss, vitiligo, and celiac disease (27). These findings are consistent with the patient’s symptoms, which included hair loss, vitiligo, and chronic diarrhea. Additionally, the patient’s low testosterone level could be attributed to testicular dysfunction caused by APS (2). The patient was admitted to the hospital due to epilepsy, and conventional antiepileptic treatment was ineffective. It is believed that the refractory epilepsy was caused by positive anti-GAD65 antibodies (33). The patient’s chronic hypokalemia is likely due to prolonged use of steroid drugs, which increase potassium ion excretion in the distal renal tubules and collecting ducts (34). It is also noteworthy that this patient had eosinophilia. While eosinophilia is more common in IPEX, its specific pathogenesis remains unclear, and it has not been previously described in APS-2.

There is currently no unified treatment plan for diseases related to positive anti-GAD65 antibodies. SPS is a progressive disease that, if not treated promptly, can lead to severe disability and even death (35). Misdiagnosis is common due to insufficient understanding by clinicians, often being mistaken for dystonia, somatic conversion disorders, motor neuron diseases, and other conditions, resulting in delayed diagnosis and treatment (36). The treatment of SPS is multifaceted, typically requiring a combination of medication (symptomatic treatment and immunotherapy) and non-pharmacological interventions. Benzodiazepine drugs, which enhance γ-GABAergic neural pathways, can improve muscle stiffness and spasms, making them the cornerstone of symptomatic treatment for SPS. Other similar drugs, such as baclofen, tizanidine, and botulinum toxin, can also be used for symptomatic treatment of SPS. Non-pharmacological interventions, including selective physical therapy (such as stretching and gait training), hyperthermia, and spinal pressure manipulation, are also effective measures for SPS treatment (37).

If symptomatic treatment and non-pharmacological interventions are ineffective, immunotherapy should be considered. Immunotherapy generally involves immunosuppressants, plasma exchange, or immunoglobulins (38). However, the optimal timing and drug selection for its intervention are not yet clear. IVIG is the most widely used and proven effective measure (38–40), with high-dose IVIG providing a stronger therapeutic response than low-dose regimens (41). Considering that the patient was already taking prednisone acetate 10mg and clonazepam 4mg, and taking into account our team’s experience with the medication and relevant studies (42–44), it was decided to treat the patient with IVIG 0.4 mg/kg for 7 days. For individuals who cannot tolerate IVIG, rituximab may be an effective alternative (45). Other immunotherapy methods, such as plasma exchange (46) and autologous hematopoietic stem cell transplantation (47), may also help improve SPS symptoms. In addition, a recent study on stiff-person syndrome with comorbid myasthenia gravis found that efgartigimod may be a candidate for the treatment of SPS and other autoantibody-mediated neurological disorders, and the research evidence provides another possibility for treating patients with anti-GAD-related disorders (48).

In this case, the patient continued to experience lower back and abdominal pain, involuntary abdominal muscle contractions, and limb stiffness despite treatment with clonazepam and steroid drugs. These symptoms significantly improved following IVIG shock therapy. This suggests that treatment with IVIG may contribute to improved outcomes in cases of APS-2 combined with anti-GAD65 antibody-related SPS. Additionally, this report is the first to note that APS-2 patients can exhibit eosinophilia, expanding the known clinical phenotype of APS-2.

However, certain limitations exist in the case data. This report highlights eosinophilia as a new phenotype of APS-2 but does not establish a direct link between this phenotype and the anti-GAD65 antibody. Future research will explore the relationship between eosinophilia and anti-GAD65 antibodies. Additionally, the patient was discharged from the hospital within a short period of time after receiving 1 time IVIG treatment, after which only remote follow-up and observation were performed, and the clinical information obtained may have been biased. At a later stage, the patient may be hospitalized for observation and, if necessary, repeat immunomodulatory treatment, according to the patient’s wishes. This will allow a more comprehensive assessment of the response and long-term prognosis of the patient after multiple IVIG treatments.

## Conclusions

In conclusion, both APS and SPS are rare clinical conditions, and their comorbidity is even rarer. Symptoms vary, the condition is often insidious, and misdiagnosis or missed diagnosis is common. Treatment regimens are complex. This article presents a rare case of APS-2 with a novel clinical phenotype combined with anti-GAD65 antibody-associated SPS. After additional treatment with IVIG, the patient’s recovery was still satisfactory, and relevant literature is reviewed, which is beneficial for improving the understanding of the disease by clinical doctors.

## Data Availability

The datasets presented in this study can be found in online repositories. The names of the repository/repositories and accession number(s) can be found in the article/**Supplementary Material**.

## Supplementary material

Supplementary Video 1 Paroxysmal spasm of the trunk muscles.
